# Supplementary material for: COZOID: contact zone identifier for visual analysis of protein-protein interactions
Source: BMC Bioinformatics. 2018 Apr 6;19:125. doi: 10.1186/s12859-018-2113-6 (PMC5889581; doi:10.1186/s12859-018-2113-6)
Supplement: Supplementary file 2 — Software build. Executable binary file of the software tool. (ZIP 118,784 kb) [file 12859_2018_2113_MOESM2_ESM.zip › cozoid/java/docs/beansbinding-1.2.1-doc.zip!/org/jdesktop/beansbinding/Binding.ValueResult.html]

Binding.ValueResult (Beans Binding)


---


|  |  |  |
| --- | --- | --- |
| **Overview** \| **Package** \| **Class** \| **Use** \| **Tree** \| **Deprecated** \| **Index** \| **Help** | |  |
| **PREV CLASS**   **NEXT CLASS** | **FRAMES**    **NO FRAMES**     **All Classes** |  |
| SUMMARY: NESTED \| FIELD \| CONSTR \| METHOD | DETAIL: FIELD \| CONSTR \| METHOD |  |


---


## org.jdesktop.beansbinding Class Binding.ValueResult<V>

```
java.lang.Object
  org.jdesktop.beansbinding.Binding.ValueResult<V>
```

**Enclosing class:**: Binding<SS,SV,TS,TV>

---

``` public static final class Binding.ValueResult<V> extends java.lang.Object ```

Encapsulates the result from calling
`Binding.getSourceValueForTarget()` or
`Binding.getTargetValueForSource()`, which
can either be a successful value or a failure.

---

| **Method Summary** | |
| --- | --- |
| `boolean` | `failed()`             Returns `true` if this `ValueResult` represents a failure and `false` otherwise. |
| `Binding.SyncFailure` | `getFailure()`             Returns the failure if this `ValueResult` represents a failure and throws `UnsupportedOperationException` otherwise. |
| `V` | `getValue()`             Returns the resulting value if this `ValueResult` does not represent a failure and throws `UnsupportedOperationException` otherwise. |
| `java.lang.String` | `toString()`             Returns a string representation of the `ValueResult`. |

**Methods inherited from class java.lang.Object**

`clone, equals, finalize, getClass, hashCode, notify, notifyAll, wait, wait, wait`

**Method Detail**

### failed

```
public boolean failed()
```

:   Returns `true` if this `ValueResult` represents
    a failure and `false` otherwise.

    :   **Returns:**: `true` if this `ValueResult` represents a failure and `false` otherwise **See Also:**: `getFailure()`

---


### getValue

```
public V getValue()
```

:   Returns the resulting value if this `ValueResult` does
    not represent a failure and throws `UnsupportedOperationException`
    otherwise.

    :   **Returns:**: the resulting value **Throws:**: `java.lang.UnsupportedOperationException` - if this `ValueResult` represents a failure **See Also:**: `failed()`

---


### getFailure

```
public Binding.SyncFailure getFailure()
```

:   Returns the failure if this `ValueResult` represents
    a failure and throws `UnsupportedOperationException`
    otherwise.

    :   **Returns:**: the failure **Throws:**: `java.lang.UnsupportedOperationException` - if this `ValueResult` does not represent a failure **See Also:**: `failed()`

---


### toString

```
public java.lang.String toString()
```

:   Returns a string representation of the `ValueResult`. This
    method is intended to be used for debugging purposes only, and
    the content and format of the returned string may vary between
    implementations. The returned string may be empty but may not
    be `null`.

    :   **Overrides:**: `toString` in class `java.lang.Object`
    :   **Returns:**: a string representation of this `ValueResult`


---


|  |  |  |
| --- | --- | --- |
| **Overview** \| **Package** \| **Class** \| **Use** \| **Tree** \| **Deprecated** \| **Index** \| **Help** | |  |
| **PREV CLASS**   **NEXT CLASS** | **FRAMES**    **NO FRAMES**     **All Classes** |  |
| SUMMARY: NESTED \| FIELD \| CONSTR \| METHOD | DETAIL: FIELD \| CONSTR \| METHOD |  |


---
